# The African Medicines Agency: historical perspective of its origins, evolution, institutional structure and future prospects

**DOI:** 10.3389/fmed.2026.1763261

**Published:** 2026-02-04

**Authors:** Alex Juma Ismail, Delese Mimi Darko, Stuart Walker, Sam Salek

**Affiliations:** 1 School of Health, Medicine and Life Sciences, University of Hertfordshire, Hatfield, United Kingdom; 2 African Medicines Regulatory Harmonisation Initiative, AUDA-NEPAD, Johannesburg, South Africa; 3 African Medicines Agency, Kigali, Rwanda; 4 Centre for Innovation in Regulatory Science, London, United Kingdom; 5 Institute for Medicines Development, London, United Kingdom

**Keywords:** Africa, African Medicines Agency, AMRH initiative, continental listing, medicine regulation, public health, regulatory harmonization

## Abstract

**Background:**

The African continent has long faced fragmented regulatory systems, resulting in delayed access to safe, effective, and quality-assured medical products. To address these challenges, the African Medicines Regulatory Harmonisation (AMRH) Programme was launched in 2009 by the African Union, laying the groundwork for the establishment of the African Medicines Agency (AMA). The AMA represents one of the most significant continental developments to harmonize regulatory practices, improve access to quality-assured medical products, and strengthen public health systems across Africa.

**Objectives:**

The objectives of this review were to examine the historical development of AMA, its Treaty and proposed institutional framework, as well as operational pilots such as the Continental Listing of Human Medicinal Products implemented by the AMRH since 2023.

**Methods:**

A narrative literature review approach was used, sourcing official African Union documents, peer-reviewed publications, and technical reports from African Union Commission, AUDA-NEPAD, WHO, and AMRH stakeholders published between 2005 and 2025.

**Results:**

The AMA was formally established by treaty adopted by the AU heads of states and governments in 2019 and entered into force in November 2021. As of June 2025, 31 AU Member States had ratified the Treaty. The agency’s governance and organizational structure include a Conference of State Parties, Governing Board, Secretariat, and Technical Committees. Pilot projects such as the AMRH Continental Listing demonstrated the feasibility of reliance mechanisms, though challenges remain in national legal harmonization, funding, and capacity disparities.

**Conclusion:**

The AMA represents a transformative step toward regulatory convergence in Africa. While challenges persist, the Treaty framework and pilot outcomes provide a strong foundation for its operationalisation and the long-term success in improving medical product regulation and public health across the continent.

## 1 Introduction

Ensuring equitable access to safe, effective, and quality-assured medicines remains one of the most pressing public health challenges across the African Continent. Despite global efforts to promote universal health coverage, African countries continue to face delayed access to essential medical products due to fragmented regulatory frameworks, limited technical capacity within National Regulatory Authorities (NRAs), resource constraints, and divergent national standards (1, 2). Fragmentation of regulatory systems across Africa has resulted in prolonged approval timelines, duplication of scientific assessments, and unequal availability of essential medicines across countries. In some cases, marketing authorisation timelines for the same generic product have differed by several years between neighbouring states, contributing to inefficiencies and delayed patient access.

Recognizing these systemic challenges, the African Union (AU), in partnership with the African Union Development Agency – New Partnerships for Africa’s Development (AUDA-NEPAD), launched the African Medicines Regulatory Harmonization (AMRH) Initiative in 2009. The Programme aimed to facilitate regional regulatory convergence by strengthening NRAs and fostering collaboration through Regional Economic Communities (RECs) (1). Early success in the East African Community (EAC) and other RECs demonstrated the feasibility of reliance-based regulatory models and joint dossier evaluations (1).

Building on the foundational work of the AMRH Programme, AU Member States formally endorsed the establishment of a continental regulatory authority, the African Medicines Agency (AMA) which was institutionalized through the AMA Treaty, adopted by the AU Assembly in February 2019 and entered into force on the 5th November 2021 following the 15th ratification (1, 3).

The AMA is envisioned as a specialized agency of the AU with a mandate to coordinate regulatory oversight across the continent, support regulatory reliance and work-sharing mechanisms, as well as harmonise the evaluation and approval of medical products. Its institutional framework includes a Governing Board, Secretariat, and specialized scientific committees such as the Evaluation of Medicinal Products Technical Committee (EMP-TC) and the Good Manufacturing Practices Technical Committee (GMP-TC) (1, 3). The AMA’s phased implementation is intended to expand Africa’s regulatory capacity, reduce duplication of regulatory efforts, and accelerate access to critical health technologies.

This manuscript provides a comprehensive review of the African Medicines Agency’s establishment. Therefore, the objectives of this work were to: (1) review the historical evolution of the AMA from the AMRH; (2) analyse the legal and institutional framework established by the AMA Treaty; and (3) examine early operational pilots including the continental listing of human medicinal products to assess the feasibility, limitations and future prospects.

## 2 Materials and methods

### 2.1 Data sources

Medline (Ovid), EMBASE, SCOPUS, and CINAHL (EBSCO) online databases from 1 January 2005 to 30 June 2023 were searched, and results corroborated by two authors (AJ, SS). Search terms included ‘African Medicines Agency’, ‘regulatory harmonisation’, ‘medical products regulation in Africa’, ‘AMRH’, ‘continental regulatory systems’, and ‘medicines regulatory authority’. Database-specific “article type/study type” filters and language limits (English) were also applied. Controlled vocabulary terms (e.g., MeSH: “Drug Regulation,” “Regulatory Agencies,” “Legislation, Drug”) and equivalent subject headings in EMBASE and CINAHL were also incorporated. Duplicates were excluded.

### 2.2 Search strategy/selection

**Figure 1:**
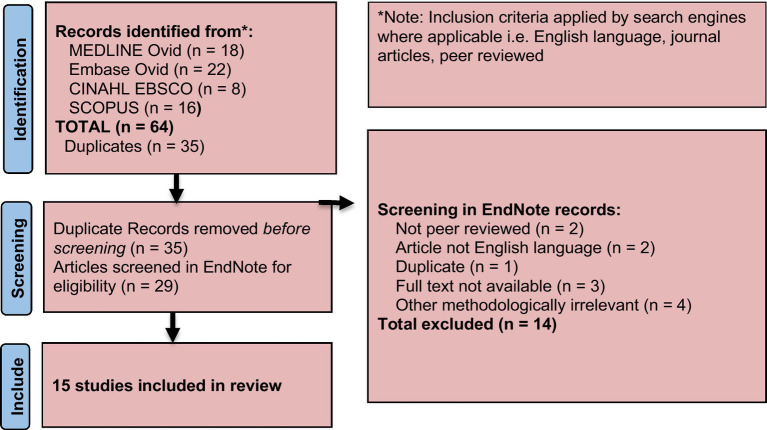
Identification, screening, and inclusion of articles.

Search results were imported into EndNote20®, to keep track of references (See references). The author AJ compared study titles and abstracts retrieved by searches against the inclusion and exclusion criteria and examined full study texts that potentially met the criteria, but whose abstracts lacked sufficient information. Rejected studies were recorded with reasoning. This process was guided by recommended methods for managing and coding references in EndNote during systematic and scoping reviews (4). Although this review was narrative in nature, selected elements of the Preferred Reporting Items for Systematic Reviews and Meta-Analyses (PRISMA) framework were applied to enhance transparency in record identification and screening; the review was not intended to meet the criteria of a full systematic review (Figure 1).

A total of 64 records were identified across all databases. After removing duplicates, titles and abstracts 29 were screened for relevance. Full texts of potentially eligible studies were then reviewed. Studies that did not meet the inclusion criteria were excluded. The final set of studies included in the narrative synthesis is presented in the Results section.

The PRISMA flow in Figure 1 refers to the selection of peer-reviewed journal articles identified through database searches. In addition, key policy, legal and technical documents from the African Union, AUDA-NEPAD, WHO, EMA, FDA and partners were included to provide context for the AMA narrative. This purposive inclusion was necessary given the institutional, legal, and policy-focused nature of the research question, which cannot be adequately addressed through peer-reviewed literature alone. These are also listed in the References.

### 2.3 Data extraction and synthesis

Data extraction was conducted using a structured pre-designed form developed for this review. For each included study, the following information was charted: author(s), year of publication, country or region, study design, regulatory function addressed (e.g., assessment, inspections, policy, governance), key findings, and relevance to the African Medicines Agency (AMA) and the African Medicines Regulatory Harmonisation (AMRH) agenda. Extracted data were independently reviewed by the two authors (AJ, SS) to ensure accuracy and consistency, with disagreements resolved through discussion.

A narrative synthesis approach was applied because of the heterogeneity in study types, regulatory themes, and analytical methods. Findings were grouped into overarching themes including: (i) evolution of regulatory harmonisation in Africa; (ii) capacity strengthening and maturity of national regulatory authorities; (iii) governance and legal frameworks relevant to AMA; and (iv) opportunities and challenges for continental regulatory systems. Patterns, gaps, convergence points, and divergences across studies were identified, and results were contextualised within the broader regulatory landscape to inform the analytical framework of this manuscript.

## 3 Results

### 3.1 Historical development of the African Medicines Agency (AMA)

The AMA was initiated out of the need to unify Africa’s fragmented regulatory environment. The AMRH Programme’s regional success in the East African Community (EAC) led to AU Executive Council endorsement in 2015 to establish a single continental regulatory body building on the foundation of the AMRH (5, 6). Between 2014 and 2019, technical working groups were established and drafted the AMA Treaty, which was adopted by AU Heads of State in February 2019 (3). The Treaty entered into force in November 2021 after reaching 15 ratifications.

The idea for a continental regulatory body first emerged formally with the launch of the AMRH Programme in 2009. AMRH sought to address challenges such as duplication of regulatory effort, variable technical standards, limited capacity and slow access to medicines. The Programme’s early success in regions like the EAC demonstrated the feasibility of harmonisation, convergence, and work sharing through joint regulatory work (1, 6). Critical milestones in the development of AMA and its legal basis are well documented, and its foundational mandate continues to evolve (1).

The development of AMA is a structured process starting in 2009, it evolved through policy endorsement, strategic planning, treaty adoption, and finally, implementation of regulatory activities. Key milestones in the evolution from AMRH to AMA are summarised in Table 1.

**Table 1:** Chronological development of AMA, 2009–2025.

Year	Event
2009	Launch of the African Medicines Regulatory Harmonization (AMRH) Programme under the AUDA-NEPAD framework. This marked the beginning of a coordinated effort to strengthen regulatory systems across Africa.
2015	AU Executive Council Decision EX.CL/Dec.857 (XXVI) officially endorses the establishment of AMA, building on the foundational work of the AMRH programme.
2015–2017	Technical Working Groups (TWGs) convened to develop the AMA concept note, business plan, and Treaty for the establishment of AMA.
2019	Adoption of the AMA Treaty by the African Union (AU) Assembly, signaling official pan-African commitment to unified regulatory oversight.
2021	Entry into force of the AMA Treaty after the required 15 AU Member States ratified the Treaty (AU, 2021).
2022–2025	AMA, through AMRH support, launches pilot operational activities such as the continental listing pilot, advancing the practical implementation of its mandate.

**Figure 2:**
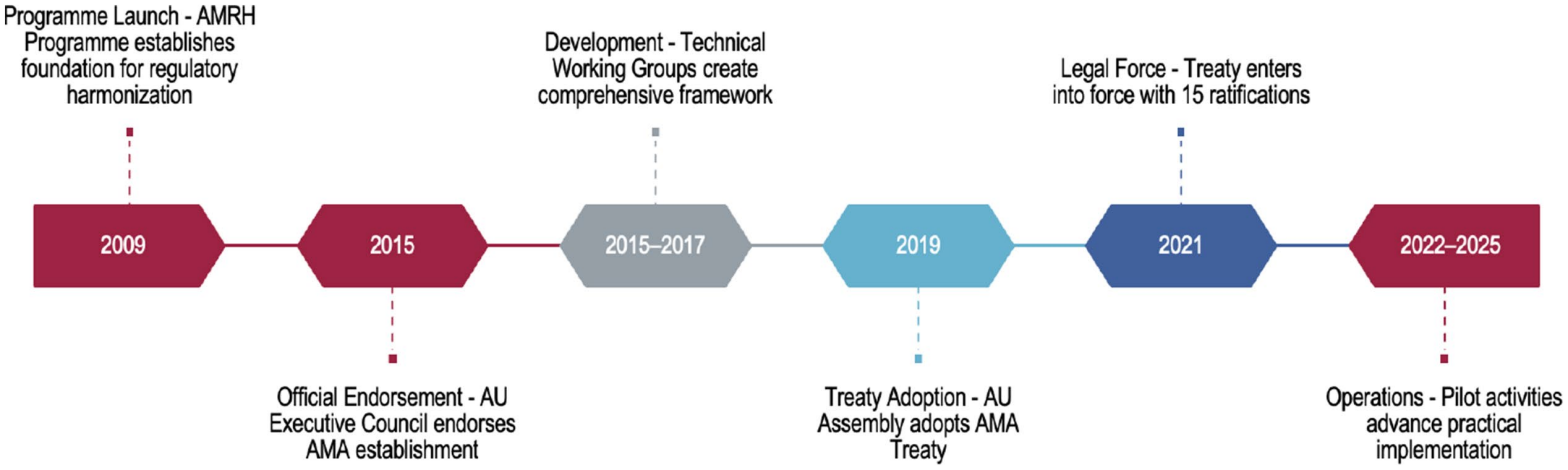
Chronological development of the African regulatory ecosystem.

These developments (also depicted visually under Figure 2) were driven by recognition that stronger, harmonized regulatory systems are essential for achieving Sustainable Development Goal 3 (Good Health and Well-being) and AU Agenda 2063 goal 3 (AU, 2030).

### 3.2 The AMA treaty: legal provisions, content and implications

The Treaty for the Establishment of the African Medicines Agency (AMA) was formally adopted by the African Union (AU) Assembly on 11 February 2019 during its 32nd Ordinary Session, held in Addis Ababa, Ethiopia (3). This Treaty provides the legal foundation for the creation of AMA as a specialized agency of the AU, with the authority to strengthen regulatory systems and coordinate medicines oversight at the continental level.

Under the Treaty, the AMA is granted international legal status, enabling it to function independently, enter into agreements, and collaborate with external partners. It is further conferred with the legal capacity to operate across AU Member States, subject to national ratification and domestication. The AMA Treaty is a legally binding international instrument for ratifying the Member States, imposing obligations related to regulatory cooperation, information sharing, and reliance mechanisms. However, implementation of AMA outputs remains contingent upon national domestication and enabling legislation, preserving national sovereignty while enabling coordinated action.

As of December 2025, thirty-one African Union Member States had ratified the Treaty for the Establishment of the AMA. Official ratification status is maintained by the AMA and published on its institutional website with corroborating records from the African Union Commission (AUC) and AUDA-NEPAD. These include early adopters such as Rwanda (which was selected to host the AMA Secretariat headquarters), Ghana, Mali, Uganda, and Seychelles. The most recent ratifications include Ethiopia (April 2024), Côte d’Ivoire (May 2024), Tanzania (April 2024), Zambia (January 2025) and Botswana (February 2025) (1). A full list of ratifying states and their dates of ratification is presented in Table 2. Additionally, it is important to distinguish between formal ratification of the AMA Treaty and technical participation in continental regulatory activities. While Table 2 summarises ratifying Member States, several non-ratifying countries continue to contribute to AMA operationalisation technical work through established work-sharing structures, including Technical Committees, joint dossier assessments, and GMP inspection collaboration under AMRH. Consequently, non-ratification should not be interpreted as non-engagement in continental regulatory harmonisation efforts.

**Table 2:** African Union Member States that have ratified the AMA treaty (as of December 2025).

No.	Country	Date of ratification
1	Algeria	06/2021
2	Benin	07/2021
3	Botswana	02/2025
4	Burkina Faso	07/2020
5	Cameroon	10/2021
6	Cape Verde	07/2023
7	Chad	10/2021
8	Côte d’Ivoire	05/2024
9	Egypt	01/2022
10	Ethiopia	04/2024
11	Gabon	10/2021
12	Ghana	03/2021
13	Guinea	05/2021
14	Kenya	07/2023
15	Lesotho	09/2022
16	Mali	06/2020
17	Mauritius	09/2021
18	Morocco	04/2022
19	Namibia	02/2021
20	Niger	08/2021
21	Rwanda	01/2020
22	Sahrawi	04/2022
23	Senegal	04/2022
24	Seychelles	11/2020
25	Sierra Leone	06/2021
26	Tanzania	04/2024
27	Tunisia	10/2021
28	Uganda	12/2021
29	Zambia	01/2025
30	Zimbabwe	09/2021
31	Togo	02/2025

African Medicines Agency (AMA), official ratification registry; African Union Commission; AUDA-NEPAD.

This table reflects ratification status of the AMA Treaty as of December 2025. Several AU Member States that have not yet ratified the Treaty may still participate technically in AMA activities (e.g., previously through AMRH Technical Committees, joint assessments, inspections, and observer participation) as provided for under Article 29 of the Treaty. Accordingly, non-inclusion in this table indicates non-ratification, not non-participation.

The mandate of the AMA, as defined by the Treaty, is to serve as the central authority for coordinating medical products regulation across Africa. This includes providing technical guidance to National Regulatory Authorities (NRAs), facilitating mutual recognition and work-sharing initiatives, and supporting the harmonization of technical standards. The AMA also aims to foster regulatory convergence among Regional Economic Communities (RECs) and global health initiatives (1, 7).

According to the Article 6 of the AMA Treaty, the core functions of AMA include:

- Harmonises medical products regulation across Africa and supports improvement of GMP inspector competence.
- Collects, manages and shares regulatory information, including data on substandard and falsified (SF) medical products.
- Coordinates joint clinical trial application reviews and supports quality control testing for countries lacking the capacity.
- Promotes and aligns regulatory policies, standards and scientific guidelines across RECs and regional health organisations.
- Designates, strengthens and oversees Regional Centres of Regulatory Excellence (RCOREs) to build regulatory workforce capacity.
- Coordinates and participates in inspections of manufacturing sites and monitors safety of medical products, sharing reports with States Parties.
- Facilitates cooperation, regulatory partnerships and mutual recognition of regulatory decisions across Africa.
- Mobilises technical and financial resources to ensure sustainability of the Agency.
- Convenes regulatory meetings like AMRC in collaboration with WHO and other partners.
- Provides regulatory guidance, scientific opinions and frameworks for action, including during public health emergencies and emerging threats.
- Advises on regulatory matters upon request from the AU, RECs or States Parties.
- Provides guidance on traditional medicines regulation.
- Advises on marketing authorisation applications for priority medicines.
- Monitors the medicines market through sample testing and shares results with countries for regulatory action.
- Develops systems to evaluate the strength and completeness of national regulatory systems and recommends improvements.
- Evaluates selected priority medical products, including complex molecules, for AU-identified priority diseases.
- Provides technical assistance and pools expertise to support countries requesting regulatory support.
- Coordinates access to and networking of national and regional quality control laboratories.
- Advocates for adoption and domestication of the AU Model Law to drive regulatory and legal reforms.

AMA’s governance architecture is composed of:

- A Conference of State Parties (CoSP) established as the highest policymaking organ of the AMA. Its core functions include setting the budget contributions from states parties; appointing or dissolving the Governing Board; establishing the rules and structure for the Director General and the Secretariat; and providing overall policy direction. It also approves the location for the headquarters and endorses RCOREs.
- A Governing Board responsible for policy direction, oversight, and strategic decision-making. Its key functions include approving strategic plans, budgets, and reports; recommending the appointment or dismissal of the Director General; and appointing the independent auditor. The Board also assists with fundraising, establishes technical committees to issue scientific guidance, and creates any subsidiary entities needed to fulfill the AMA’s mission, as tasked by the CoSP.
- A Secretariat led by Director General which manages day-to-day operations, technical coordination, and inter-agency collaboration.
- Technical Committees established by the Governing Board as either permanent or *ad hoc* to provide technical guidance on specific areas of regulatory expertise. They handle all core scientific work. They review product dossiers and clinical trials, inspect manufacturing facilities and provide the scientific opinions needed for the AMA to function. These committees also carry out any additional tasks assigned to them by the Governing Board.

Membership in the AMA is open to all AU Member States that have signed and ratified the Treaty. However, the AMA may also cooperate with non-ratifying states on specific technical issues, under observer or associate frameworks.

The AMA Treaty represents a major legal and political step in institutionalizing a unified regulatory mechanism across Africa, positioning AMA as a central pillar in the continent’s response to future pandemics, health emergencies, and pharmaceutical quality challenges is critical as concerns regarding national sovereignty have featured prominently in discussions surrounding the establishment of the African Medicines Agency. Unlike supranational regulatory authorities, the AMA does not issue legally binding marketing authorisations. Instead, it provides coordinated scientific opinions and facilitates reliance mechanisms that support national decision-making while preserving statutory authority at the country level. In this respect, the AMA represents a hybrid regulatory model. While the European Medicines Agency (EMA) operates under a shared sovereignty framework using common legislative instruments in which centrally authorised products are legally binding across European Union Member States and for example organisation like the PAHO functions as a cooperative platform supporting national regulators in the Americas, the AMA is tailored to Africa’s legal heterogeneity by combining continental coordination with voluntary national implementation. This design reflects the realities of varying legal systems, regulatory maturity, and political contexts across African Union Member States, enabling progressive convergence without undermining national accountability.

To contextualize the AMA’s institutional and functional design, it is helpful to compare it with that of other major global regulatory agencies. Table 3 presents a side-by-side comparison between AMA, the European Medicines Agency (EMA), and the United States Food and Drug Administration (FDA) highlighting the distinct legal bases, reliance mechanisms, and scopes of regulatory authority across these agencies.

**Table 3:** Comparative overview: AMA vs. EMA vs. US FDA.

Feature	AMA (Africa Union)	EMA (European Union)	FDA (United States)
Legal basis	Treaty for the Establishment of the African Medicines Agency (2019): A binding treaty adopted by the AU Assembly under its Constitutive Act. AMA operates as a specialized agency with international legal status, subject to national ratification and domestication	EU Regulations and Directives: Primarily governed by Regulation (EC) No 726/2004 and Directive 2001/83/EC. Regulations are directly applicable across EU states, while Directives require national implementation. Legal authority stems from the Treaty on the Functioning of the EU.	Federal Food, Drug, and Cosmetic Act (FDCA, 1938): A U.S. federal statute granting FDA full authority to regulate human and veterinary drugs, biologics, food, and medical devices. Regularly amended, e.g., by the 21st Century Cures Act.
Jurisdiction	Operates in 31 AU Member States (as of October 2025) that have ratified the Treaty. Participation is voluntary and requires signing, ratification and domestication.	Covers all 27 EU Member States under a shared sovereignty model, where regulatory decisions are binding across the Union.	Covers the entire United States, including states and territories. FDA regulations are federally binding and enforced nationwide.
Product scope	Covers all types of medical products as defined by AU Model Law (e.g., medicines, vaccines and other biologics, diagnostics as well as non-IVDs medical devices. The treaty also covers traditional medicines and emerging technologies).	Focuses mainly on human and veterinary medicinal products including vaccines. Some roles in advanced therapy medicinal products (ATMPs).	Broad remit over pharmaceuticals, biologics, devices, food, dietary supplements, and cosmetics.
Approval mechanism	Based on continental listing (Article 6): AMA coordinates evaluation and listing of approved products to support national reliance strategies. AMA does not issue binding approvals but facilitates national marketing authorization through work sharing models.	Utilizes a centralized approval procedure where the EMA evaluates and grants EU-wide marketing authorization for certain medicines. Decentralized, Mutual recognition as well as national routes also exist.	FDA grants full centralized federal approvals for all products entering the U.S. market. Mandatory review and enforcement across product lifecycle.
Emergency use provisions	Treaty for its establishment allows technical committees under AU mandate to recommend emergency listings or use authorizations. Processes still evolving.	Permits conditional marketing authorizations during public health emergencies (e.g., pandemics), often with real-world evidence requirements.	Grants Emergency Use Authorizations (EUA) under national emergency declarations, allowing unapproved products to be used during crises (e.g., COVID-19).
Technical support role	Provides capacity building, technical guidelines, GMP/GLP inspections, training, and regulatory convergence for Regional Economic Communities and National Regulatory Authorities (NRAs), especially in low-resource countries.	Offers scientific advice, regulatory guidance, and convergence support for member states. EMA plays a key role in regulatory science development in the EU.	Conducts pre-market review, manufacturing inspections, post-market surveillance, and enforcement. Provides extensive regulatory guidance and compliance support.

The AMA model draws selectively from international precedents while avoiding authoritative regulatory centralisation. By emphasising reliance, work-sharing, and mutual recognition rather than compulsory harmonisation the AMA seeks to balance efficiency gains with respect for national sovereignty, a consideration that has been critical to securing Treaty ratification and political support. The above comparative framework (Table 3) illustrates AMA’s unique positioning as a hybrid model of decentralised coordination and technical harmonisation, tailored to Africa’s legal diversity and public health imperatives.

### 3.3 Administration and institutional framework of AMA

The institutional design of the African Medicines Agency (AMA) reflects the ambition to build a strong, technically robust, and continentally coordinated regulatory authority for medical products in Africa. As stipulated in the Treaty for the Establishment of AMA (Article 10), the Agency is composed of four core organs: the Conference of State Parties, Governing Board, the Secretariat, and Technical Committees (3).

- The Conference of the States Parties (CoSP) established as the highest policymaking body of the AMA, is composed of all African Union Member States that have ratified the Treaty, each represented by a minister responsible for health or their duly authorised representatives. The CoSP convenes at least once every two years, with provisions for extraordinary meetings, and is responsible for overseeing implementation of the Treaty, electing the Governing Board, confirming the appointment of the Director General, adopting rules of procedure, approving work programs and budgets, as well as guiding the AMA’s strategic policy direction.
- The Governing Board functions as the primary executive organ of the African Medicines Agency (AMA), entrusted with providing strategic oversight, approving regulatory and technical guidelines, and monitoring organizational performance. It is composed of members appointed by the Conference of the States Parties including five Heads of National Regulatory Authorities from 5 AU recognized regions, one representative of RECs responsible for regulatory harmonisation, one representative of regional health organisation (RHO), One Representative of National Committees Responsible for Bioethics and the Commissioner for Social Affairs at the African Union Commission. Members are appointed on a rotational basis as per criteria set out in the AMA Treaty.
- The Secretariat functions as the AMA’s administrative and operational hub. It is responsible for day-to-day management, technical coordination, engagement with National Regulatory Authorities (NRAs), and implementation of the AMA programs. The Secretariat is headed by a Director-General, who is appointed by the Governing Board and confirmed by the CoSP. The Director-General oversees the execution of all activities and supervises the overall work of the Agency as its Chief Executive Officer.
- Technical Committees established by the Governing Board as either permanent or *ad hoc* to provide technical guidance on specific areas of regulatory expertise. The committees are established in dossier assessment for advanced therapies, biologicals (including biosimilar and vaccines); medicines for emergencies, orphan medicinal products; clinical trials of medicines and vaccines; manufacturing site inspections of Active Pharmaceutical Ingredients (API) and finished pharmaceutical products, quality control laboratories; bioavailability and bioequivalence studies; pharmacovigilance risk assessment; and African traditional medicines.

#### 3.3.1 Phased implementation strategy

To support an orderly and cost-efficient operationalization of the Agency, AMA will be implemented in three distinct phases: Foundation (2024–2025), Expansion (2026–2030), and Maturity (2031 onward) as outlined in the 2025 AMA Organizational Plan approved by its Governing Board and adopted with implementational changes by the AMA Conference of State Parties (8).

##### 3.3.1.1 Foundation phase (2024–2025)

This phase emphasizes establishing AMA’s essential regulatory, administrative, and governance structures. It includes:

- Recruitment of 43 core team of essential staff, including the Director-General’s Office, administrative operations, and key technical and regulatory personnel.
- Development and adoption of operational guidelines, information systems, and quality assurance frameworks.
- Formation and adoption of functional coordination mechanisms between AMA and NRAs, RECs, and global partners.
- Initiation of internal systems such as finance, IT, procurement, legal compliance, and partner engagement.
- Monitoring of timelines and Regulatory Performance

This phase is designed to ensure AMA can begin functioning while laying the groundwork for more advanced regulatory services to the continent.

##### 3.3.1.2 Expansion phase (2026–2030)

The Expansion Phase is focused on scaling AMA’s technical operations, formalizing work-sharing models, and strengthening cross-border collaboration. Key features include:

- Recruitment of an additional 150 staff from the foundation phase.
- Take over and launch advanced regulatory functions including evaluation and marketing authorisation, vigilance, safety monitoring, GMP and Good Clinical Practices inspections including emergency use assessments and listings.
- Development and revisions of harmonized regulatory templates, assessment reports, inspection guidelines, and technical training modules.
- Establishment and operationalisation of digital platforms for a continental regulatory process.

This phase positions AMA as the central reference point for regulatory reliance across Africa, while supporting national capacity development and convergence.

##### 3.3.1.3 Maturity phase (2031 onward)

The final phase envisions AMA operating at full capacity as a continental and global center of regulatory excellence. By this stage, AMA is expected to:

- Coordinate all Treaty-mandated functions, including lifecycle regulation, post-marketing surveillance, quality control, and policy development.
- Support mutual recognition of regulatory decisions among States Parties.
- Maintain robust regional and international partnerships (e.g., with AUDA-NEPAD, Africa CDC, WHO, EMA and US FDA).
- Operate a harmonized and integrated regulatory systems linked with national pharmacovigilance platforms.

#### 3.3.2 Operational budget and costing

According to the 2025 AUC costed implementation plan, AMA’s staff budget for the Foundation Phase (8 months of operations in 2025) is estimated at USD 7.57 million, covering personnel, recruitment, allowances, training, medical and welfare expenses. This phased costing approach ensures sustainability and enables the Agency to attract donor support and AU member contributions during its early operational years.

The institutional and operational design of AMA reflects a carefully sequenced strategy rooted in the Treaty’s provisions and informed by continental and global regulatory models. Through its phased implementation, the Agency is expected to evolve into a fully operational, technically credible, and politically supported mechanism for regulatory harmonization across Africa.

#### 3.3.3 Financing and sustainability

The financial sustainability of the AMA is guided by its Treaty and envisaged as a phased model combining State Parties contributions, transitional partner support, and progressive cost-recovery mechanisms for services rendered to industry. During the foundation phase, AMA operations are supported through assessed contributions from ratifying AU Member States, supplemented by development partners to enable start-up functions, infrastructure development, and capacity building.

In the medium to long term, the AMA is expected to introduce activity-based cost-recovery mechanisms aligned with regulatory services, such as dossier assessments, inspections, and scientific advice, drawing on international best practices while remaining sensitive to the economic diversity of its Member States.

### 3.4 The role of Regional Economic Communities (RECs) in the establishment and operationalisation of the AMA

RECs have served as the cornerstone for medicines regulatory harmonisation in Africa, providing both structural and technical platforms upon which the African Medicines Agency (AMA) can effectively build. Under the African Medicines Regulatory Harmonisation (AMRH) initiative, five RECs were formally recognised as “Regional Economic Committees (RECs)” tasked with coordinating joint assessments, inspections, and capacity-building activities across Member States (9). These RECs including, the East African Community (EAC), the Southern African Development Community (SADC), the Economic Community of West African States (ECOWAS), the Economic Community of Central African States (ECCAS), and the Intergovernmental Authority on Development (IGAD) each developed their own technical working groups and Standard Operating Procedures (SOPs) for dossier evaluation, inspection, and post-market surveillance.

#### 3.4.1 Foundational structures and workstreams

By mid-2023, AMRH’s implementation leadership model comprised ten Technical Committees (TCs) working in tandem with five RECs. Technical Committees are intentionally multidisciplinary and may co-opt *ad hoc* experts in clinical pharmacology, disease-specific disciplines, and emerging technologies as required. Engagement is envisaged through Regional Centres of Regulatory Excellence (RCOREs), collaboration with academic institutions, and partnerships with WHO technical networks. Each REC oversaw a legal framework or “Model Law” adaptation exercise, aligning national legislation with a continental standard to facilitate mutual recognition of regulatory decisions (9). The harmonised guidelines produced by RECs for quality-, safety and efficacy-assessment of medical products including vaccines and complex biologics formed the template upon which the AMA would later expand. For example, the EAC’s Joint Assessment Procedure (EAC-MRH) provided over a decade of lessons on synchronising multi-country dossier reviews, demonstrating that pooled expertise reduces duplication of effort and shortens approval timelines (9).

#### 3.4.2 Capacity building and training platforms

Each REC has organized regular workshops and train-the-trainer programs for NRA personnel covering topics such as Good Review Practices (GReVP), good manufacturing practice (GMP) inspections, and pharmacovigilance. These training programmes created a cadre of harmonised experts familiar with a common set of performance indicators and process metrics (10). When the AMA came into force, these REC-trained experts were readily “redeployed” into the AMA’s core teams, ensuring that continental review sessions and joint inspections could be conducted without delay. Indeed, the Treaty for the Establishment of the AMA explicitly states that one of AMA’s primary objectives is to “enhance capacity of State Parties and AU-recognized RECs to regulate medical products in order to improve access to quality, safe and efficacious medical products” (3).

#### 3.4.3 Legal and policy harmonisation and reliance

The RECs led the transposition of the AU Model Law on Medical Products Regulation into national legislations, thereby creating legal interoperability among Member States. This laid the groundwork for AMA’s envisaged legal mandate to conduct joint reviews that would one day be “automatically recognised” by all ratifying countries. By mid-2023, for instance, at least three RECs had established Memoranda of Understanding (MoUs) among their Member States’ NRAs to recognise each other’s inspection and assessment report an approach explicitly endorsed in the AMA Treaty as the “reliance mechanism” (3).

#### 3.4.4 Piloting joint assessments and information-sharing systems

Long before AMA’s formal entry into force in November 2021, the RECs had begun piloting continent-wide information-sharing platforms (e.g., waVAPI in ECOWAS, MRH portal in EAC). These electronic registries allowed participating NRAs to upload assessment reports, inspection findings, and safety alerts in near real time thereby streamlining communication across borders. AMA’s Information and Communications Technology (ICT) strategy now simply scales these REC-piloted systems into a unified continental database.

#### 3.4.5 Regional advocacy and mobilisation of member states

The RECs played a key advocacy role in mobilizing AU Member States to sign and ratify the AMA Treaty. By using regular REC-level ministerial forums (e.g., EAC Health Ministers’ Council meetings), harmonisation champions highlighted both public-health benefits and economic incentives (e.g., pooled procurement, streamlined trade under the AfCFTA). As a result, by June 2023, 35 out of 55 AU Member States had either signed or ratified the Treaty largely through REC-supported advocacy (9).

In summary, RECs have provided AMA with pre-existing governance structures, harmonised guidelines, trained human capital, ICT platforms for information sharing, and a legal-policy foundation. As AMA transitions to full operationalisation, it will build directly upon these REC achievements shifting from a pilot-by-pilot approach to a single, integrated continental agency.

### 3.5 The role of National Regulatory Authorities (NRAs) in the establishment and operationalisation of the AMA

While the RECs offer the macro-level regulatory harmonisation framework, National Regulatory Authorities (NRAs) remain the “frontline engines” that anchor AMA’s work in each country. The AMA Treaty (Articles 4, 5 and 6) explicitly stipulates that the Agency shall not supplant NRAs, but rather coordinate and facilitate joint regulatory activities with the objective of strengthening national systems (3). In practice, AMA functions are designed around pooled expertise and reliance mechanisms which allow NRAs to retain statutory authority while benefiting from continental coordination. This model is intended to enhance, rather than deplete, national regulatory capacity, particularly in resource-constrained settings. Practically this means the following aspects.

#### 3.5.1 Ensuring readiness through institutional strengthening

The AMA’s effectiveness hinges on each NRA’s capacity to perform baseline regulatory functions dossier screening, risk-based inspections, laboratory testing, and pharmacovigilance. As Ngum et al. (9) conclude, “an effective AMA will need strong National Medicines Regulatory Authorities as well as Regional programmes.” Over the past decade, the following NRAs namely Egypt, Ethiopia Ghana, Nigeria, Rwanda, Senegal, South Africa, Tanzania & Zimbabwe have used the World Health Organization’s (WHO) Global Benchmarking Tool (GBT) to raise their maturity level to at least “Level 3,” thereby making them eligible to participate fully in joint review procedures (10).

#### 3.5.2 Participation in joint assessment and joint inspection procedures

The NRAs nominate technical staff to serve on AMA’s Assessment Pool and Inspection Teams. During the pilot phase, six NRAs Kenya, Uganda, Rwanda, Tanzania, Zanzibar, and Burundi participated in the East African Community’s Joint Assessment Procedure (EAC-MRH). These NRAs agreed to share both workload and data: each country would perform a full scientific assessment of a dossier, then circulate the findings to other participating NRAs for “concurrence.” The AMA has adopted this same model at a continental scale, inviting all ratified NRAs to either lead an assessment or serve as a “reliance partner” when their own capacity is limited (9).

#### 3.5.3 Alignment of Good Review Practices (GReVP)

Good Review Practices (GReVP) serve as the “rulebook” that standardises how dossiers are evaluated, how questions are communicated to applicants, and how final decisions are documented. Nancy Ngum et al. (10) showed that among the EAC-MRH Member States, variability in GReVP directly correlated with approval timelines and consistency of decisions. Consequently, the AMA’s Technical Guidelines require each NRA to adopt a uniform set of GReVP (e.g., standardized templates for summaries of scientific reviews, decision letters, and risk-assessment frameworks). The NRAs should then undergo periodic “peer reviews” to assess adherence to these practices.

#### 3.5.4 Legal harmonisation at the national level

To effectively operationalise the African Medicines Agency (AMA), each NRA must domesticate the African Union (AU) Model Law on Medical Products Regulation by incorporating its provisions into national statutes. This legal transposition is essential for enabling the AMA to conduct joint regulatory activities such as dossier assessments and joint inspections that are legally binding and automatically recognised within individual countries. By mid-2025, 31 NRAs had enacted enabling legislation or provisions that allow for delegated functions to AMA, such as the registration of generic products through reliance pathways or shared marketing authorizations (1, 11, 12). This harmonised legal foundation is critical for ensuring regulatory consistency, accelerating access to quality-assured medical products, and reinforcing the regional impact of AMA’s decisions

#### 3.5.5 Contributing to the continental risk-profiling and post-market surveillance system

To strengthen post-authorization monitoring across the continent, each NRA is required to contribute to pharmacovigilance (PV), data on substandard and falsified (SF) medical products and Adverse Drug Reactions (ADRs) to a continental surveillance database managed by the African Medicines Agency (AMA). Most NRAs currently utilize the WHO’s VigiBase or national electronic pharmacovigilance platforms, which feed into AMA’s real-time signal detection system. This regional mechanism enhances early warning capabilities and fosters collaborative risk assessment. Crucially, NRAs must also concede certain data-sharing controls to the AMA Secretariat thereby allowing safety alerts identified in one jurisdiction to trigger rapid, continent-wide regulatory action for product recalls, label updates, or market withdrawals. This harmonised model mirrors best practices in global pharmacovigilance and is essential for ensuring public health security in a pan-African context.

#### 3.5.6 Sustaining financial and human-resource commitments

Although the AMA is a continental agency, each NRA remains financially responsible for seconding staff and providing logistical support to joint assessment sessions. As of June 2023, participating NRAs were assessed on a “cost-sharing” model whereby low- and middle-income countries received differential AMA Secretariat subsidies to cover travel and daily subsistence allowances for their technical experts (9). This model incentivizes NRAs to invest in capacity building so that they can “pay their own way” in future joint activities.

#### 3.5.7 Coordination with other national stakeholders

Effective AMA participation requires that NRAs liaise with national Ministries of Health, local Good Manufacturing Practice (GMP) inspectors, and national pharmacovigilance centers. For instance, a joint inspection of API facility in South Africa will involve South Africa’s NRAs, local GMP auditors, and the national Pharmacovigilance Programme of India counterpart ensuring that AMA-led inspections dovetail seamlessly with domestic regulatory actions.

### 3.6 The role of other stakeholders for AMA sustainability

AMA’s long-term effectiveness hinges not only on technical strength but also on robust stakeholder engagement.

#### 3.6.1 Key stakeholder roles

The AMA’s long-term sustainability depends on engagement beyond just the regulatory authorities. Civil society and patients groups plays a critical role in advocating for transparency, accountability, and equitable access to medical products. Academia contributes through regulatory science research, training, and disease-specific expertise while industry engagement is essential for compliance, dossier quality, and post-marketing surveillance. Development partners support capacity building, financial sustainability, and infrastructure development.

## 4 Operationalization of AMA: snapshot of the pilot of continental listing of human medicinal products

This section provides a high-level snapshot of the Continental Listing pilot conducted under the AMRH as a preparatory step toward AMA operationalisation. The focus is on describing the institutional set-up, procedural flow, and observed feasibility of reliance-based regulatory coordination.

The African Medicines Regulatory Harmonisation Programme launched a Continental Listing Pilot in August 2023 to test and validate AMA’s regulatory procedures. This initiative was implemented by the Evaluation of Medicinal Products Technical Committee (EMP-TC) and the Good Manufacturing Practices Technical Committee (GMP-TC) the two primary scientific structures designated to perform pre-approval evaluation and inspections for medicinal products across Africa (1).

**Figure 3:**
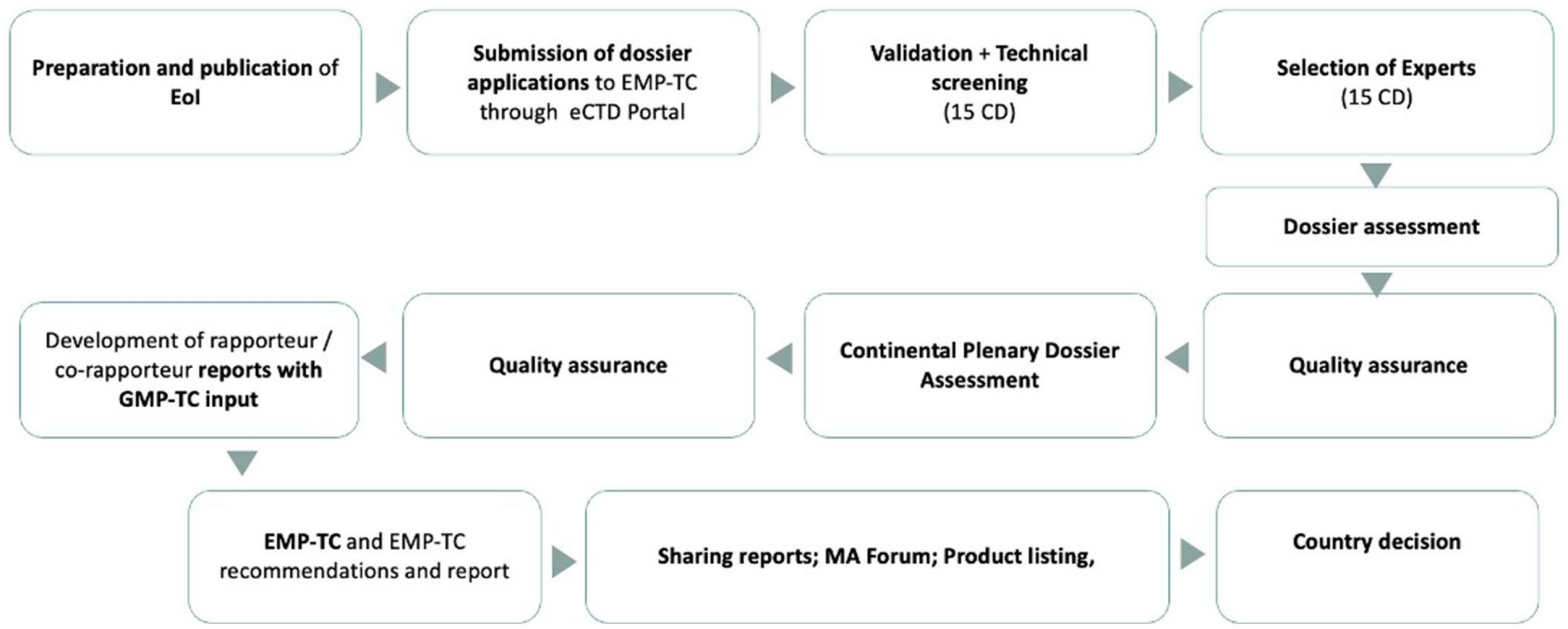
Continental pathway for listing of human medicinal products.

The pilot was formally endorsed by the AMRH Steering Committee following the adoption of the continental assessment procedure during the 9th African Medicines Regulators Conference (AMRC). Its objective was to trial the evaluation and inspection procedures for priority products and assess how NRAs could rely on these outputs for national marketing authorisations. Figure 3 shows a summarized continental pathway followed during the pilot phase.

The pathway illustrates the delineation of responsibilities across institutional levels, with the AMRH Secretariat coordinating process management, EMP-TC and GMP-TC providing scientific assessment and inspection outputs, and NRAs retaining final national decision-making authority.

### 4.1 Scope and participation

A call for Expressions of Interest (EOIs) was launched on 1 November 2023, inviting manufacturers to submit dossiers for priority medical products aligned with the eligibility criteria approved by the EMP-TC. In response, the AMRH Secretariat received 64 EOIs from 18 manufacturers, covering over 20 different manufacturing sites of drug substances and drug products. From these, 24 medicinal products were shortlisted to participate in the pilot after which 3 products were removed from the pilot during cycle one of evaluation based on non-conformity with terms and conditions for participating in the pilot phase.

The pilot incorporated both desktop and on-site assessments, with 78 assessors and 72 inspectors objectively selected by EMP-TC and GMP-TC and officially endorsed and appointed by their respective AU Member States to participate. These professionals underwent onboarding training and were deployed as primary reviewers, peer reviewers, lead Inspectors, co-inspectors or QA experts. EMP-TC and GMP-TC members served as rapporteurs, co-rapporteurs, or lead inspectors under the standard operating procedures of the AMRH Continental Listing framework (1).

During the pilot phase, product labelling, lifecycle management, and country-specific adaptations remained under the authority of NRAs. The continental listing provided a scientific opinion to support national decisions, while post-authorisation activities continued to follow national legal and public health requirements.

### 4.2 Evaluation and inspection activities

As part of the Continental Listing Pilot, each medicinal generic product dossier underwent a clearly defined multi-stage evaluation process aligned with the African Medicines Agency’s (AMA) emerging regulatory procedures. The process began with technical screening via the SAHPRA-hosted DocuBridge platform (an electronic dossier submission and review system), where applications were reviewed for completeness and formatting conformity. Eligible dossiers then advanced to first, second and third multi-level-cycles of scientific assessments, coordinated by the Evaluation of Medicinal Products Technical Committee. These assessments were completed well within the AMRH benchmark of 100 days, 60 days and 30 days for first, second and third cycle of product review, respectively.

In parallel, GMP inspections were executed by the GMP Technical Committee as part of the continental pilot. A total of 24 GMP inspections were conducted, comprising 17 onsite inspections, 3 virtual inspections, and 4 desk reviews. All inspections were performed using the harmonised procedures, checklists, and reporting templates from the AMRH GMP Playbook, ensuring consistency and comparability across sites.

The inspected manufacturing facilities were located in India, Italy, Egypt, Ireland, Switzerland, the Netherlands, and the United States, reflecting the global footprint of the pilot and confirming AMA’s capability to coordinate regulatory oversight across multiple jurisdictions.

By 31 October 2025, 12 medicinal products had successfully received a positive scientific opinion and were added to the Continental List of Human Medicinal Products (Green Book), 2 negative opinion and rejected and 7 products deferred by the Steering Committee to the next phase of continental listing to be considered under AMA. These products passed through harmonised multi-cycle evaluations, with the AMRH technical timelines ranging from 148 to 162 days and total times to listing between 194 and 208 calendar days, all within the AMRH overall target of 210 days. Throughout the pilot, assessment and inspection activities were conducted using harmonised templates, standard operating procedures, and quality assurance checkpoints developed under the AMRH framework. Independent quality assurance reviews were applied to assessment reports prior to plenary consideration, reinforcing consistency and scientific robustness across multinational review teams. These milestones demonstrate the operational viability of the EMP-TC and GMP-TC, and the broader feasibility of collaborative, cross-border regulatory processes for product evaluation and facility inspection (12). Detailed quantitative performance analyses, including comparative timeline reductions, efficiency metrics, and programme-level outcomes, are the subject of a separate programme-level evaluation of the Continental Listing pilot and are therefore not examined in depth in this institutional review.

### 4.3 NRA reliance and outcomes

The reliance experiences described below are illustrative and intended to demonstrate feasibility rather than to provide a comprehensive comparative analysis of national uptake. The pilot confirmed that several NRAs were willing and able to adopt AMA recommendations via reliance mechanisms, with Cote Dvoire, Tanzania, Ghana and Zambia reportedly approving a product within 2, 10 and 21, 28 working days, respectively, following either the EMP-TC recommendation or continental listing decision. Other NRAs required additional national steps or faced internal procedural barriers before full reliance could be implemented.

Feedback from both regulators and industry participants acknowledged the pilot’s value in enhancing technical consistency, reducing duplicative evaluations, and facilitating access to products faster. However, stakeholders also highlighted the need for procedural clarity, particularly in:

- Post-listing national decision timelines
- Requirements for sample submissions
- Management of lifecycle activities such as post-approval variations.

### 4.4 Lessons learned

The pilot yielded a number of key insights:

- Technical screening exceeded expectations with some dossiers processed in as little as 1–2 days, compared to the 15-day benchmark.
- Language barriers and translation (e.g., French to English) introduced delays in a few dossiers.
- EMP-TC meeting frequency was a bottleneck; reliance on virtual plenaries meant applications sometimes waited several weeks before discussions.
- Some NRAs pre-emptively registered products before continental listing finalisation, contrary to the proposed reliance framework.

These findings emphasize the need for stronger communication protocols, enhanced digital systems, and increased harmonisation of national procedures with continental timelines. They also directly informed the refinement of AMA operating procedures, technical committee workflows, and guidance documents adopted during the foundation phase of AMA implementation.

### 4.5 Conclusion

The 2023 pilot demonstrated the technical feasibility and operational value of a continental reliance-based product listing system. It laid the groundwork for formalizing AMA’s regulatory operations, validated the tools and SOPs for use in full implementation, and identified clear areas for improvement. Going forward, these lessons are being used to revise guidance documents, inform capacity-building strategies, and structure AMA’s full regulatory function rollout under its Foundation Phase.

## 5 Challenges identified during the establishment of the AMA

Despite strong political will across the African Union (AU), the development and operationalization of the African Medicines Agency have encountered multifaceted legal, technical, and institutional challenges. These obstacles became especially apparent during both the ratification of the AMA Treaty and the implementation of the 2023 Continental Listing Pilot, revealing structural weaknesses in national systems, regulatory harmonization, and infrastructure (12). These challenges and enabling factors have been widely documented across regulatory harmonisation literature, economic evaluations, and policy analyses in Africa (15–30).

### 5.1 Legal and sovereignty barriers

A primary barrier is the lack of a harmonized legal framework among AU Member States. Although the AU Model Law on Medical Products Regulation was adopted in 2016 to support domestic reforms, a 2019 review revealed that only five countries had fully domesticated the law, with 13 others in progress (12). Without enabling legislation that allows reliance on the AMA decisions or joint inspections, many NRAs remain bound to conduct full national reviews slowing product approvals and diminishing the AMA’s utility.

Moreover, sovereignty concerns persist in some Member States. Regulatory bodies have expressed reluctance to delegate authority to AMA due to institutional traditions, legal ambiguity, and concerns about political accountability, creating friction in treaty implementation (12, 13).

### 5.2 National domestication gaps

While 31 AU Member States have ratified the AMA Treaty as of 2025, only a subset has enacted enabling provisions that allow national regulators to formally rely on AMA outputs. This partial domestication has led to duplicated assessments, delayed national approvals, and unclear procedures for post-listing activities like sample submissions or labeling updates (12).

### 5.3 Capacity disparities and regulatory maturity

Significant country-level variation persists in regulatory capacity across African Union Member States, reflecting differences in financial resources, institutional maturity, and historical investment in medicines regulation. While several middle-income countries such as South Africa, Egypt, Ghana, and Rwanda have attained WHO Maturity Level 3 (14). Many low-income countries continue to operate at earlier weak stages of regulatory development, with limited human resources, under-resourced pharmacovigilance systems, and reliance on manual or semi-digital processes.

These disparities directly influence the extent to which NRAs can engage in, and rely upon, AMA-coordinated regulatory outputs. NRAs with higher maturity levels are more likely to assume rapporteur or lead inspector roles, whereas lower-capacity authorities often participate as reliance partners or observers. Without targeted capacity-strengthening interventions, there is a risk that uneven implementation of reliance mechanisms could reinforce existing regulatory asymmetries rather than reduce them. The AMRH initiative has provided support through WHO’s Global Benchmarking Tool (GBT) Institutional Development Plans (IDPs) support, however, closing the gap across all jurisdictions remains a long-term goal that AMA will take forward.

### 5.4 Digital infrastructure and data sharing

The absence of interoperable digital platforms across NRAs presents another major challenge. There is inconsistent uptake of systems like electronic Common Technical Document (eCTD) or DocuBridge, which hampers cross-country dossier exchange and inspection coordination. Some NRAs also face issues with cybersecurity and data confidentiality key requirements for regulatory reliance (Lorenz, n.d., unpublished technical briefing)^1^. The AMA’s proposed Continental Regulatory Information Management System (RIMS) aims to address these weaknesses, but it requires significant investment and integration support (1).

### 5.5 Resource mobilization and sustainability

Many NRAs struggle with limited funding and staffing, hindering their participation in reliance-based assessments. The absence of a harmonized cost recovery model such as differentiated fees for AMA-reliant approvals further discourages engagement. This lack of incentives, combined with the absence of predictable approval timelines, makes it difficult for resource-constrained NRAs to adopt the AMA outputs in practice (1).

### 5.6 Operational uncertainty and transition gaps

Several NRAs have expressed confusion regarding post-pilot procedures, especially concerning the operational steps after receiving an AMA scientific opinion. Issues include uncertainty around sample submissions, variations and renewals, and the synchronization of labeling decisions. In the absence of a formalized AMA–NRA coordination protocol, many authorities reverted to full national reviews undermining the efficiency gains from continental listing (13).

A consolidated overview of the key challenges and their corresponding implications for AMA implementation is presented in Table 4.

**Table 4:** Key challenges in the development and implementation of the African Medicines Agency (AMA).

Challenge Area	Description	Implications for AMA implementation
1. Legal and sovereignty barriers	Fragmented national legal systems; slow domestication of the AU Model Law; concerns over regulatory sovereignty	Hinders reliance-based decision-making; delays mutual recognition; weakens legal authority of AMA outputs
2. Treaty domestication gaps	AMA Treaty ratified by many states but not translated into enabling national legislation	Prevents formal legal reliance on AMA decisions; prolongs national registration timelines
3. Capacity disparities	Wide variation in regulatory maturity (e.g., WHO GBT ratings); limited trained assessors and inspectors	Undermines trust in joint decisions; limits implementation of harmonized technical standards
4. Digital infrastructure deficits	Lack of integrated platforms for submission, review, tracking, and cross-border data sharing	Impairs efficiency of joint reviews, delays access to shared regulatory information
5. Resource mobilization gaps	Absence of sustainable funding models or cost-recovery mechanisms for AMA participation	Constrains technical engagement by underfunded NRAs; risks unequal participation
6. Operational uncertainty	Unclear post-listing protocols (e.g., lifecycle management, sample requirements, labeling coordination)	Leads to inconsistent national adoption; duplication of work; undermines continued effort.

Addressing these disparities will require differentiated implementation strategies, including targeted training, digital infrastructure support, and transitional reliance models tailored to the specific needs of low-income and less mature regulatory systems.

## 6 Future prospects for the African Medicines Agency

With the AMA Treaty ratified by 31 Member States as of December 2025 and AMA Headquarters now operational in Kigali, Rwanda, the African Medicines Agency is strategically positioned to become a cornerstone of Africa’s evolving health architecture. The Agency holds transformative potential to address long-standing inefficiencies in medical product regulation by coordinating regulatory harmonization, improving reliance, and enhancing health systems integration across the continent.

### 6.1 Accelerating access to quality medical products

The AMA is designed to reduce duplication of regulatory assessments and streamline marketing authorisation pathways across Africa. By establishing a Continental Listing of Human Medicinal Products and facilitating mutual reliance, the AMA enables countries to issue national approvals based on central scientific opinions. This significantly shortens time-to-market for essential medicines and vaccines; a capability particularly critical for pandemic preparedness and timely response to public health emergencies.

By enabling reliance-based regulatory decisions and coordinated scientific assessments, the AMA has the potential to substantially reduce approval timelines for vaccines, essential medicines, and emergency treatment products. This is particularly critical in public health emergencies, where delayed regulatory action can exacerbate morbidity and mortality. The continental coordination facilitated by AMA supports equitable access by reducing duplication, improving regulatory predictability, and enabling faster national uptake of quality-assured products, thereby strengthening Africa’s collective health security.

Participation in the 2023 AMRH pilot already demonstrated that countries such as Tanzania and Ghana could complete reliance-based decisions within days of a continental recommendation, compared to traditional processes that take several months.

### 6.2 Enhancing public health surveillance and emergency response

The AMA’s future role includes supporting pharmacovigilance systems, post-marketing surveillance, and emergency use authorisations (EUAs). Article 18 of the AMA Treaty outlines the establishment of Scientific Committees with mandates to issue technical opinions on safety, efficacy, and quality, including during health crises. These committees may serve as the backbone of regional and continental response systems by pooling scientific expertise and aligning procedures across countries.

The AMA’s coordination with WHO, the Africa Centres for Disease Control and Prevention (Africa CDC), and NRAs could allow it to function as a regulatory rapid-response mechanism during future outbreaks, ensuring that essential health products are reviewed, authorized, and distributed swiftly.

### 6.3 Supporting local pharmaceutical manufacturing

Through regulatory harmonization and predictable approval pathways, AMA can support local manufacturers in gaining faster access to continental markets. By eliminating redundant evaluations and creating uniform standards for Good Manufacturing Practices (GMP) and inspections, the AMA lowers regulatory barriers for African producers, enabling them to compete regionally and globally.

This alignment complements the goals of the Pharmaceutical Manufacturing Plan for Africa (PMPA) and the African Continental Free Trade Area (AfCFTA) by linking local production with harmonized regulation, pooled procurement, and trade facilitation.

### 6.4 Driving regulatory convergence and global partnerships

The AMA is well-positioned to become a globally recognized continental regulatory authority, similar to the European Medicines Agency (EMA) or the Pan American Health Organization (PAHO). It fosters regulatory convergence by serving as a central technical reference point for NRAs and Regional Economic Communities (RECs). Its model promotes the recognition of trusted assessments, enables work-sharing, and contributes to the international dialogue on regulatory science.

The AMA is also expected to strengthen partnerships with:

- World Health Organization (WHO): to support NRAs in achieving WHO Maturity Level 3.
- European Medicines Agency (EMA): through technical exchange, inspector training, and reliance frameworks.
- African CDC and Global Donors: for coordinated emergency use pathways and health security infrastructure

### 6.5 Institutionalizing sustainability and stakeholder engagement

To sustain momentum, the AMA must embed mechanisms for:

- Predictable funding and cost recovery based on differentiated fee structures.
- Digital infrastructure such as the Continental Regulatory Information Management System (RIMS) for dossier tracking, pharmacovigilance, and lifecycle management.
- Industry and civil society engagement platforms to ensure transparency and responsiveness to stakeholder needs.

Continued political commitment, strategic investment in regulatory workforce development, and strong partnerships will be vital for the AMA to realize its full potential as a pan-African regulatory institution. To illustrate these emerging roles and strategic linkages. Table 5 summarises the AMA’s core future functions alongside relevant partners, highlighting anticipated areas of coordination across the continental regulatory ecosystem.

**Table 5:** Strategic future roles of the African Medicines Agency (AMA).

Strategic area	AMA function/potential role	Key partners/linkages
Access to medical products	Coordinate continental assessment and listing of medicines and vaccines	NRAs, RECs, WHO, UNICEF, Africa CDC and GAVI
Emergency preparedness and response	Provide emergency use authorizations and joint inspection mechanisms during public health crises	Africa CDC, WHO, AUDA-NEPAD, CEPI
Surveillance and safety monitoring	Strengthen vigilance, post-market surveillance, and adverse event reporting systems for medical products	AUDA-NEPAD, Africa CDC, National PV Centres, WHO, Uppsala Monitoring Centre
Support for local manufacturing	Streamline regulatory pathways for African manufacturers	Africa CDC, AfCFTA, RECs, AUDA-NEPAD and WHO
Regulatory convergence	Promote mutual recognition, work-sharing, and alignment of technical standards	WHO, EMA, ICH, IMDRF, GHWP and Africa CDC
Training and capacity building	Facilitate training, assessor certification, and joint inspection	WHO, RECs, Africa CDC, AUDA-NEPAD
Global regulatory positioning	Serve as a continental regulatory voice and partner in international regulatory fora	ICH, WHO, EMA, IMDRF, GHWP, US FDA
Sustainability and innovation	Implement cost-recovery mechanisms, digital systems (RIMS), and lifecycle regulatory management	AU Member States, Donors, Bill and Melinda Gates Foundation

UNICEF, United Nations Children’s Fund; GAVI, the vaccine alliance; CEPI, coalition for epidemic preparedness innovations; ICH, international council for harmonisation; IMDRF, international medical device regulators forum; GHWP, global harmonization working party.

### 6.6 AMA’s Post-2030 role

Beyond 2030, the AMA is expected to operate at full maturity as a continental centre of regulatory excellence. Its role is anticipated to expand from coordination of pre-market evaluation to encompass lifecycle regulation, post-marketing surveillance, emergency use authorisations, and advanced regulatory science functions. As national legal domestication progresses, AMA’s outputs are expected to be increasingly relied upon by Member States, reinforcing regulatory convergence while preserving national decision-making authority.

## 7 Conclusion

This review demonstrates that the AMA represents a landmark institutional innovation aimed at addressing long-standing fragmentation in Africa’s regulatory landscape. The AMA Treaty provides a robust legal foundation, while early operational pilots through AMRH have demonstrated the feasibility of reliance-based continental regulation. Nonetheless, challenges related to legal domestication, capacity disparities, financing, and digital infrastructure remain significant. Addressing these constraints will be critical to ensuring the AMA’s long-term effectiveness and legitimacy.

To maximise impact, policy priorities should include accelerated ratification of the AMA Treaty, sustained investment in regulatory capacity, development of predictable financing mechanisms, and continued engagement with national and regional stakeholders. If effectively implemented, the AMA has the potential to transform access to quality-assured medical products and strengthen public health outcomes across the African continent.

Without doubt, the AMA represents a landmark institutional innovation in Africa’s journey toward regulatory harmonisation, improved access to quality-assured medical products, and strengthened public health systems. Rooted in the policy vision of the African Union and operationalized through the foundations laid by the AMRH initiative, AMA is poised to unify a continent long fragmented by diverse regulatory standards and capacities.

This manuscript has reviewed the Agency’s historical evolution, the legal framework provided by the AMA Treaty, its envisaged organizational structure, phased implementation strategy, and the operational insights gained through the 2023–2025 Continental Listing pilot. Together, these elements illustrate both the feasibility and the promise of AMA as a vehicle for regulatory convergence, reliance, and institutional capacity development. Despite this promise, significant challenges remain. Legal inconsistencies among AU Member States, limited domestic capacity in many NRAs, inadequate digital infrastructure, and uncertainty around AMA’s scientific work and sustainability models must all be addressed to ensure the AMA’s long-term impact. The pilot experience highlighted both the effectiveness of reliance-based assessments and the procedural complexities that can arise in the absence of harmonised national frameworks.

Looking forward, the AMA’s success will hinge on sustained political commitment, strategic investment in regulatory science, and the ability to integrate its functions with global regulatory systems and regional health initiatives. Its role in facilitating continent-wide joint reviews, rapid response during public health emergencies and support for local pharmaceutical manufacturing places the AMA at the heart of Africa’s health sovereignty agenda. If fully implemented and supported, the AMA has the potential not only to streamline regulatory systems within Africa, but to become a globally recognized center of regulatory excellence and continental counterpart to entities like the EMA. Its success will ultimately be measured by its ability to translate scientific rigor, political unity, and institutional innovation into tangible health outcomes for over 1.4 billion people across the African continent.

This historical perspective has led to the following recommendations:

1. Evaluate the continental regulatory outcomes and limitations of the AMRH pilot project for the listing of human medicinal products. This includes documenting the implementation process, success factors, and operational barriers encountered.
2. Analyze the structure, performance, and lessons from the Evaluation of Medicinal Products Technical Committee (EMP-TC) and Good Manufacturing Practices Technical Committee (GMP-TC), drawing comparisons with the European Medicines Agency committee model.
3. Assess stakeholder perspectives (including NRAs, RECs, the pharmaceutical industry, and development partners) on the legitimacy, feasibility, and effectiveness of the continental regulatory framework and mechanism tested under the pilot.
4. Evaluate the contributions of the Coordination and Implementation Platform of partners involvement and engagement (CIP and AMRH PP) in supporting technical operations, financial planning, and educational development relevant to AMA’s operationalisation.
5. Propose a functional secretariat organisation and technical committee working style for the AMA, including mechanisms for engaging non-ratifying AU member states through Memoranda of Understanding (MoUs).
6. Conduct an economic evaluation of AMRH’s regulatory activities and propose a sustainable fee structured model for AMA informed by lessons from the pilot, international best practices and benchmarking with EMA, WHO-PQ, and select African NRAs.

## Footnotes

1 Lorenz J. Digital regulatory information management systems and data governance challenges in medicines regulation in Africa. Unpublished technical briefing; African regulatory harmonisation context (n.d.).

## Glossary

ADR: Adverse Drug Reaction
AfCFTA: African Continental Free Trade Area
AMA: African Medicines Agency
AMRC: African Medicines Regulators Conference
AMRH: African Medicines Regulatory Harmonisation
API: Active Pharmaceutical Ingredient
AU: African Union
AUC: African Union Commission
AUDA-NEPAD: African Union Development Agency–New Partnership for Africa’s Development
CEPI: Coalition for Epidemic Preparedness Innovations
CoSP: Conference of State Parties
DG: Director-General
EAC: East African Community
ECCAS: Economic Community of Central African States
ECOWAS: Economic Community of West African States
eCTD: electronic Common Technical Document
EMA: European Medicines Agency
EMP-TC: Evaluation of Medicinal Products Technical Committee
EOI: Expression of Interest
EUA: Emergency Use Authorization
FDA: U.S. Food and Drug Administration
GAVI: Gavi, the Vaccine Alliance
GBT: (WHO) Global Benchmarking Tool
GHWP: Global Harmonization Working Party
GMP: Good Manufacturing Practice
GMP-TC: Good Manufacturing Practice Technical Committee
GReVP: Good Review Practices
ICH: International Council for Harmonisation
IDP: Institutional Development Plan
IGAD: Intergovernmental Authority on Development
IMDRF: International Medical Device Regulators Forum
MoU: Memorandum of Understanding
NRA: National Regulatory Authority
PMPA: Pharmaceutical Manufacturing Plan for Africa
PV: Pharmacovigilance
REC: Regional Economic Community
RCORE: Regional Centre of Regulatory Excellence
RIMS: Regulatory Information Management System
SADC: Southern African Development Community
SDG: Sustainable Development Goal
SF: Substandard and Falsified (medical products)
SOP: Standard Operating Procedure
TC: Technical Committee
TWG: Technical Working Group
UMC: Uppsala Monitoring Centre
UNICEF: United Nations Children’s Fund
WHO: World Health Organization
WHO PQ: WHO Prequalification
